# Fluorescence-based *in situ* assay to probe the viability and growth kinetics of surface-adhering and suspended recombinant bacteria

**DOI:** 10.1186/1559-4106-8-22

**Published:** 2013-08-21

**Authors:** Ima Avalos Vizcarra, Philippe Emge, Philipp Miermeister, Mamta Chabria, Rupert Konradi, Viola Vogel, Jens Möller

**Affiliations:** 1 Department of Health Sciences and Technology, Laboratory of Applied MechanobiologyETH ZurichCH-8093ZurichSwitzerland; 2 Fraunhofer Institute for Manufacturing Engineering and Automation IPANobelstraße 1270569StuttgartGermany; 3 BASF SE, Advanced Materials and Systems ResearchD-67056LudwigshafenGermany

**Keywords:** Antimicrobial surfaces, Optical viability monitoring, Green fluorescent protein (GFP), SYTO^®^ 9, Propidium iodide (PI)

## Abstract

Bacterial adhesion and biofilm growth can cause severe biomaterial-related infections and failure of medical implants. To assess the antifouling properties of engineered coatings, advanced approaches are needed for *in situ* monitoring of bacterial viability and growth kinetics as the bacteria colonize a surface. Here, we present an optimized protocol for optical real-time quantification of bacterial viability. To stain living bacteria, we replaced the commonly used fluorescent dye SYTO^®^ 9 with endogenously expressed eGFP, as SYTO^®^ 9 inhibited bacterial growth. With the addition of nontoxic concentrations of propidium iodide (PI) to the culture medium, the fraction of live and dead bacteria could be continuously monitored by fluorescence microscopy as demonstrated here using GFP expressing *Escherichia coli* as model organism. The viability of bacteria was thereby monitored on untreated and bioactive dimethyloctadecyl[3-(trimethoxysilyl)propyl]ammonium chloride (DMOAC)-coated glass substrates over several hours. Pre-adsorption of the antimicrobial surfaces with serum proteins, which mimics typical protein adsorption to biomaterial surfaces upon contact with host body fluids, completely blocked the antimicrobial activity of the DMOAC surfaces as we observed the recovery of bacterial growth. Hence, this optimized eGFP/PI viability assay provides a protocol for unperturbed *in situ* monitoring of bacterial viability and colonization on engineered biomaterial surfaces with single-bacteria sensitivity under physiologically relevant conditions.

**Electronic supplementary material:**

The online version of this article (doi:10.1186/1559-4106-8-22) contains supplementary material, which is available to authorized users.

## Background

Clinically relevant nosocomial infections are frequently caused by adherent bacteria and the subsequent biofilm formation within tissues or on biomaterial surfaces [1]. Surface biofouling commonly starts with the adhesion of individual bacteria that subsequently grow into mature biofilms. To prevent bacterial adhesion and growth already during the pre-biofilm phase, two main surface engineering strategies have been developed so far: non-fouling “stealth” surface coatings that inhibit adhesion of proteins and bacteria [2–4] and bioactive materials, which upon bacterial contact or release of the active molecules interfere with bacterial viability [5–10]. To compare the antimicrobial properties of surface coatings and to study the kinetics of bacterial surface colonization, assays are needed that allow for *in situ* monitoring of bacterial adhesion and viability. The gold standard for bacterial viability tests has long been quantification of colony forming units (CFU) by plating bacterial suspensions that were incubated with the test surface on nutrient agar [11]. Counting bacterial colonies, which result from plating suspended viable and cultivatable bacteria, however, does not account for the inherent phenotypic heterogeneity and the ability of the bacteria to persist in dormant states [12, 13]. Furthermore, plating assays lack the ability to measure the colonization and viability kinetics directly on the test surface and might not be representative for the surface-attached bacterial population.

**Figure 1:**
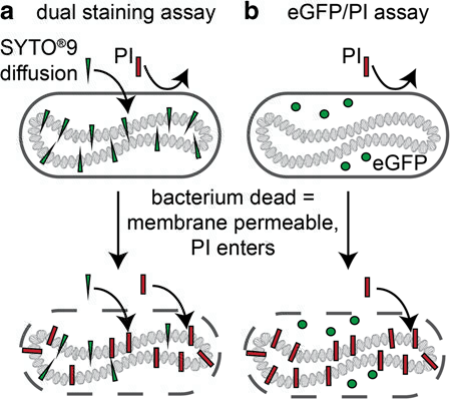
**Bacterial viability assays in comparison. (a)** Conventional *endpoint* dual staining bacterial viability assay and **(b)** eGFP/PI assay optimized for *in situ* bacterial viability monitoring. The dual staining assay commonly employs two DNA stains, SYTO^®^ 9 (green triangle) and propidium iodide (PI, red rectangle) that both intercalate into the bacterial DNA. SYTO^®^ 9 diffuses passively into living bacteria whereas PI cannot pass intact bacterial membranes and only enters permeabilized dead bacteria. The use of constitutive eGFP expression rather than SYTO^®^ 9 provides direct detection of viable bacteria without the addition of a fluorescent dye and circumvents the competitive displacement of SYTO^®^ 9 by PI that can result in a dual staining of dead bacteria.

An alternative to determine bacterial viability is to probe for the bacterial membrane integrity that is maintained by energy-dependent processes in living bacteria and is lost upon bacterial death [14]. Membrane integrity can be tested optically by using a combination of membrane permeable and impermeable fluorescent dyes that selectively enter live and dead bacteria (Figure 1a) [8, 14–17]. While being broadly employed as endpoint staining assays to determine the viability of single bacteria and bacterial colonies directly on the test surface, these assays are not optimized for real-time *in situ* bacterial viability monitoring. Particularly when DNA intercalating dyes like SYTO^®^ 9 and propidium iodide (PI) are used [18], the impact of the potentially toxic stains on bacterial physiology has to be considered to avoid false negative results [19]. Furthermore, since both stains target DNA, the competitive displacement of the SYTO^®^ 9 (live stain) by the high affinity PI (dead stain) upon membrane breakdown can affect the staining reliability [20]. To eliminate the competitive displacement of the two DNA stains and the demand for prolonged incubation times caused by the passive diffusion of the SYTO^®^ 9 live stain through the bacterial membrane, it was suggested to replace SYTO^®^ 9 with green fluorescent protein (GFP) expressed by the bacteria as demonstrated previously for flow cytometry applications (Figure 1b) [21]. Although flow cytometry has been used to measure the viability of GFP expressing bacteria adsorbed to polystyrene beads functionalized with antimicrobial coatings [22], it cannot be applied for continuous *in situ* bacterial viability monitoring on planar surfaces.

Building upon those observations, we present an optimized protocol to probe the viability and growth kinetics of surface-adhering and suspended bacteria using non-toxic concentrations of propidium iodide and *Escherichia coli* that express the fluorescent protein GFP. Beyond calibrating the assay and monitoring *E. coli* surface colonization kinetics on bare glass substrates, we demonstrate that this assay is applicable to monitor the inactivation kinetics of *E. coli* in contact with antimicrobial surface coatings, using dimethyloctadecyl[3-(trimethoxysilyl)propyl]ammonium chloride (DMOAC) coated glass surfaces as model substrate. To kill bacteria, the quaternary ammonium chloride complexes of surface-bound DMOAC have to directly interact with the bacterial membrane [23]. We previously showed that bacterial fimbriae strongly influence the unspecific adhesion of *E. coli* to engineered surfaces [4]. Type 1 fimbriae (7 nm diameter, several 100 μm length) protrude from the bacterial membrane thereby preventing the bulk bacterial body from direct interaction with the underlying material surface. To ensure a physical contact of the bacterial membrane with the material surface, we used here the non-fimbriated K-12 derivative AAEC191A *E. coli* strain. In addition, we highlight the effect of serum protein adsorption on the bactericidal properties of antimicrobial surfaces. We incubated the DMOAC surfaces with fetal bovine serum (FBS) to mimic the physiological situation where serum proteins adsorb to engineered biomaterials upon contact with host body fluids.

## Methods

### Bacteria

Non-fimbriated *E. coli* AAEC191A bacteria, a derivative of *E. coli* K-12 MG1655 containing a deletion in the entire *fim* cluster [24] was provided by Prof. E. Sokurenko, University of Washington, Seattle, USA. For GFP expression, chemocompetent AAEC191A *E. coli* were transformed with eGFP pHis plasmid under the control of the *tac* promoter (AAEC191A pHis-GFP). To obtain *E. coli* that express eGFP under the control of the constitutive rpsm promoter (AAEC191A rpsm-GFP), *E. coli* AAEC191A were transformed with the rpsm-GFP plasmid that was extracted from the original fusion library strain MG1655 rpsm-GFP [25] by Qiaprep Spin Miniprep kit (Qiagen 27106). Transformed bacteria were selected by cultivation on LB agar plates supplemented with either 100 μg/ml ampicillin (pHis-GFP) or 50 μg/ml kanamycin (rpsm-GFP). Bacterial precultures were inoculated from glycerol stocks into LB medium (5 g/l yeast extract, 10 g/l tryptone, 10 g/l NaCl) containing appropriate antibiotics. To induce GFP expression in AAEC191A pHis-GFP *E. coli*, 0.1 mM Isopropyl-β-D-thiogalactopyranoside (IPTG, Applichem A1008) was added. LB precultures were grown overnight at 37°C under continuous shaking at 180 rpm (Infors Unitron HT). To ensure defined culture conditions for the bacterial growth and viability assays, bacteria from the overnight culture were centrifuged at 1700 g, washed three times and subcultured in 20 ml minimal M9 medium (1x M9 salts (Sigma-Aldrich M6030), 10 mM Mg_2_SO_4_ (Sigma-Aldrich 63126), 10 g/l Glucose (Sigma-Aldrich G8270), 0.5 mM CaCl_2_ (Sigma-Aldrich C5080), 1x MEM vitamins (Gibco 11120), 1x MEM amino acids (Gibco 11130)) supplemented with the appropriate antibiotics and 0.1 mM IPTG for AAEC191A pHis-GFP *E. coli*. Bacteria were subcultured at 37°C, 180 rpm until exponential growth phase (OD_600_ = 0.3-0.8). Bacteria were harvested by centrifugation at 1700 g followed by three washing steps. Immediately before the experiment, bacteria were resuspended in M9 medium that contained the strain-specific antibiotics, IPTG as well as 0, 3, 30 μM PI (Sigma-Aldrich, 81845) and 6 μM SYTO^®^ 9 (Invitrogen L13152), respectively.

### Growth curve measurements

Growth curves of suspended bacteria were recorded by turbidity measurements at 600 nm in 96 well plates (Tecan Infinity 200 Pro plate reader). Kinetic measurements were performed every 15 minutes at 37°C and continuous shaking. Bacteria were inoculated to an initial turbidity of 0.01 at 600 nm in M9 medium containing appropriate antibiotics, PI and SYTO^®^ 9.

### Viability assay

For kinetic viability measurements under physiological conditions, bacteria were cultivated in an ibidi^®^ glass bottom flow chamber (ibidi, 80168) within a temperature-controlled microscope incubator to guarantee constant nutrient supply and optimal growth conditions at 37°C. Bioactive surfaces were prepared according to published protocols [23]. Briefly, glass cover slides, that later resemble the bottom slide of the flow chamber, were exposed to air plasma for 15 seconds (Harrick Plasma, PDC-32G) followed by dipping into a 5% (v/v) aqueous DMOAC (Sigma-Aldrich) solution for 1 second, and drying at 105°C overnight. To test the effect of protein pre-incubation on the antimicrobial activity of the DMOAC coatings, slides were incubated in undiluted fetal bovine serum (FBS, Thermo Scientific SH30071.02) for 1 h prior to the assembly of the flow chamber. Bare glass cover slides were attached to the ibidi^®^ chambers as control surfaces. The bacterial suspension (OD_600_ 0.05) in M9 medium containing different concentrations of PI and SYTO^®^ 9, was directly added to the flow chamber and immediately transferred to an epifluorescence microscope (Nikon TE2000-E) for *in situ* viability monitoring. Adhesion of bacteria to the glass bottom slide was allowed for 5 minutes before the flow chamber was gently washed with 5 ml M9 medium (flow rate 0.01 ml/min) to remove non-adherent bacteria. As control staining at defined time points, the *Bac*Light™ viability kit (Invitrogen, L13152) was used according to the supplier instructions.

### Ellipsometry

The adsorbed dry film thickness of DMOAC and DMOAC + FBS layers on silicone wafers was measured by variable-angle spectroscopic ellipsometry (VASE) using the M2000F variable-angle spectroscopic ellipsometer (J.A. Woollam Co., Inc.). The measurement was performed at 70° relative to the surface normal under ambient conditions. Ellipsometry data were fitted using a cauchy model with parameters for organic layers (n(λ) = Aλ + Bn/λ^2 + Cn/λ^4, with An = 1.45, Bn = 0.01, Cn = 0.0) to obtain dry thickness of adlayers.

### Image segmentation and quantification

To limit the viability analysis to fluorescent *E. coli* and to eliminate bias in the data analysis based on GFP fluorescence intensity, fluorescence images were thresholded and segmented using the morphological strel algorithm of the image processing toolbox of MATLAB^®^ software (MATLAB, MathWorks; version R2010b) that combines image erosion and dilation operations. The algorithm was included into a semiautomatic image processing workflow that allows for manual adjustment of the thresholding levels of the entire time series as well as individual time frames. Binary masks were generated from the thresholded images and surface-adherent bacteria were counted automatically. The summing of binary masks from consecutive time points allowed for correction of fluorescence signal loss caused by GFP bleaching, washout and degradation of the stained DNA. To prevent false-positive results, binary masks that were not positive for the GFP channel before, were excluded from the PI positive counts to limit the analysis to bacteria that were viable initially. Elimination of x,y drift of time series data was achieved by the register virtual stack slices and transform virtual stack slices plugins of Fiji that were incorporated into a MATLAB^®^ routine using the MIJ java package for bi-directional communication between MATLAB^®^ and ImageJ by D. Sage. The MATLAB^®^ file for the analysis workflow is available in the Additional file 1.

## Results

### Impact of SYTO^®^ 9 and propidium iodide concentrations on the growth of suspended *E. coli*

**Figure 2:**
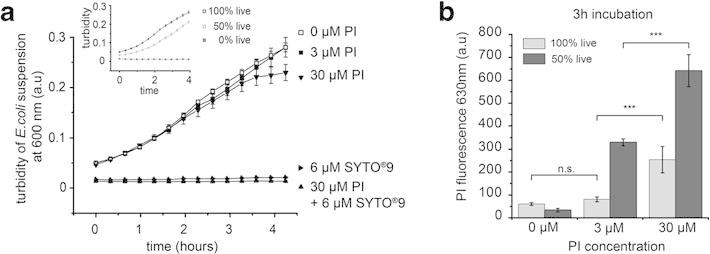
**Impact of SYTO**^**®**^**9 and propidium iodide (PI) concentration on*E. coli*growth rate and the detection efficiency of dead bacteria in M9 bacterial medium. (a)** Inhibition of bacterial growth in the presence of 6 μM SYTO^®^ 9 and 30 μM PI. PI alone showed a dose-dependent growth inhibition. At a concentration of 30 μM *E. coli* growth was inhibited, which was not detected when the PI concentration was reduced tenfold from 30 to 3 μM. Replication of *E. coli* in 3 μM PI containing M9 medium was confirmed by growth rate measurements from a starting culture of 50% live / 50% dead *E. coli* (inset) **(b)** PI fluorescence of *E. coli* cultivated in M9 medium containing different PI concentrations. 3 μM PI sufficiently stained dead bacteria in a 50% live / 50% dead *E. coli* mixture, while no significant background signal increase was detected for 3 μM PI compared to the background for a 100% live bacterial solution. In contrast, supplementing the medium with 30 μM PI resulted in a significant increase of PI stained bacteria from a 100% live starting culture indicating that this high concentration of the DNA stain interferes with bacterial viability. Background fluorescence of PI supplemented M9 medium was subtracted for each of the three PI concentrations, respectively. Error bars represent the standard error of the mean.

To determine toxicity levels of the fluorescent DNA stains, we probed the effect of SYTO^®^ 9 and propidium iodide (PI) on *E. coli* growth. We supplemented bacterial batch cultures in M9 growth medium with varying dye concentrations (0, 3, 30 μM PI, 6 μM SYTO^®^ 9) and monitored bacterial growth at 37°C by quantifying the increase in the turbidity of the solution at 600 nm (Figure 2a). 6 μM SYTO^®^ 9 in combination with 30 μM PI, as recommended in the conventional and commercial dual staining assay [26], completely inhibited *E. coli* growth. Supplementing the *E. coli* suspensions with 6 μM SYTO^®^ 9 alone showed the same growth inhibition, while 30 μM PI itself did not inhibit growth completely but did reduce the growth rate compared to the pure M9 medium. This indicates that 6 μM SYTO^®^ 9 causes major changes in bacterial physiology. The impaired growth rate upon addition of PI was eliminated when we decreased the PI concentration in the bacterial growth medium tenfold, i.e. from 30 μM to 3 μM (Figure 2a).

To confirm that the viable *E. coli* in the medium supplemented with 3 μM PI were able to replicate, we compared the turbidity increase of a 50% live / 50% isopropanol killed bacterial mixture to cultures containing 100% live and 100% isopropanol treated *E. coli* (Figure 2a). Within 4 hours, the turbidity increase for the mixed 50% live / 50% dead starting culture did not reach the same level as for the 100% live culture. Those results are consistent with the expected exponential growth rate of viable bacterial batch cultures and thus show that the bacteria replicated normally in 3 μM PI containing medium. To determine if the reduced PI concentration was sufficient to detect dead bacteria in solution, we incubated bacterial batch cultures starting from either 100% live or 50% live / 50% killed bacteria in M9 medium containing 3 and 30 μM PI respectively. The PI fluorescence at 630 nm was subsequently measured by fluorescence spectroscopy over 3 hours (Figure 2b). Supplementing the growth medium with 3 μM PI adequately stained the isopropanol treated bacteria in the 1:1 mixture of live and dead *E. coli* but did not result in a significant increase of the background fluorescence of the 100% live starting culture. In contrast, supplementing the medium with 30 μM PI significantly increased the PI fluorescence from the 100% live starting culture (Figure 2b), which was consistent with the impaired growth rate under those conditions (Figure 2a), indicating that 30 μM but not 3 μM PI is toxic to *E. coli* bacteria.

### Viability and growth rate of surface-adhering *E. coli* is strongly reduced upon long-term incubation in culture medium supplemented with SYTO® 9

**Figure 3:**
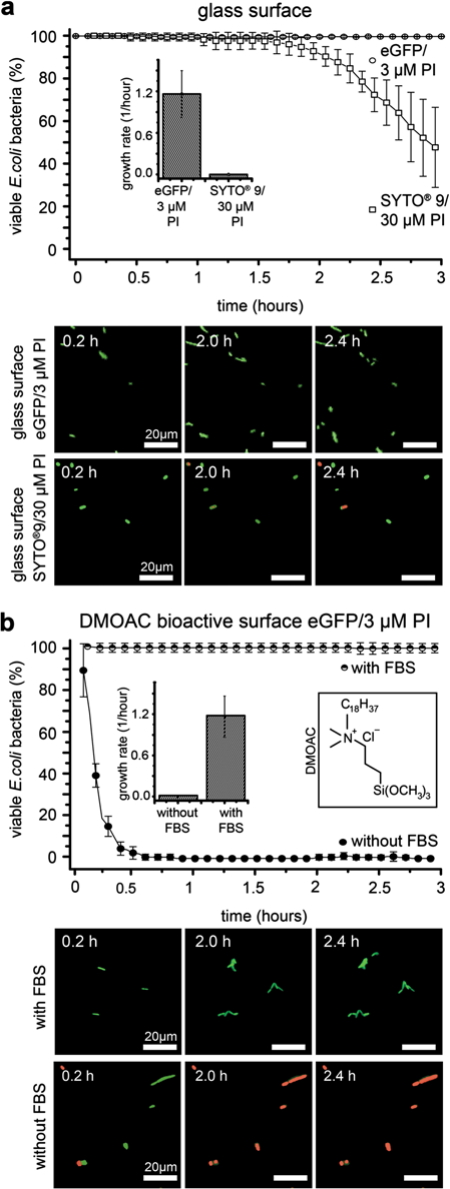
***In situ*monitoring of the growth and viability of surface-adhering*E. coli*using the eGFP/3 μM PI assay. (a)** Time series of surface-adhering *E. coli* on bare glass substrates. The dual staining assay (6 μM SYTO^®^ 9 / 30 μM PI) decreases bacterial viability on untreated glass substrates after incubation times longer than 1 hour as *E. coli* (AAEC191A) incubated with 6 μM SYTO^®^ 9 containing medium failed to replicate (inset). In contrast, eGFP-expressing *E. coli* (AAEC191A pHis-GFP) that were incubated with 3 μM PI were able to replicate and grow on the glass surface. **(b)** Viability of *E.coli* (AAEC191A pHis-GFP) on antimicrobial DMOAC-coated glass surfaces as monitored by eGFP/PI fluorescence microscopy. Pre-exposure of the DMOAC surfaces to fetal bovine serum (FBS) completely blocked the antimicrobial activity. Microscopy images show the overlay of the SYTO^®^ 9 / eGFP and PI fluorescence channels, i.e. differentiating live (green) from dead bacteria (red). 3 independent fields of view from different experiments were analyzed containing a total of 125–250 surface attached bacteria for each condition. Error bars represent the standard deviation. Scale bar 20 μm.

**Figure 4:**
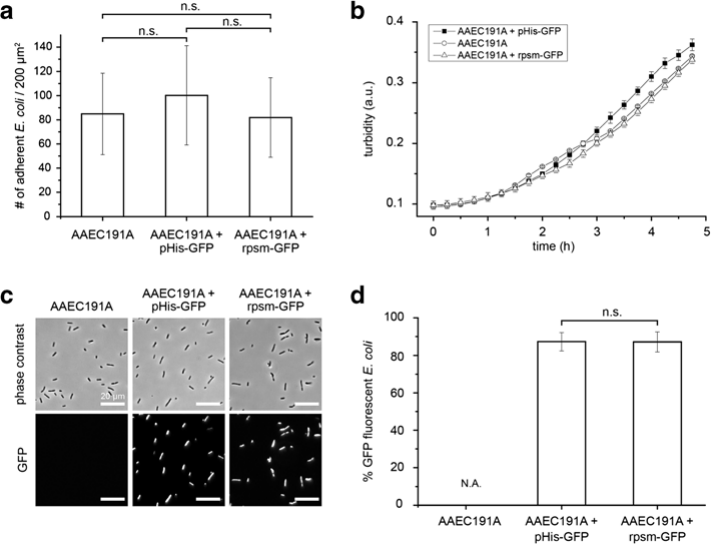
**Influence of GFP expression on the adhesion and fluorescence of*E. coli*K-12 derivative strains. (a)** The number of *E. coli* adhering to bare glass substrates was analyzed for the non-fimbriated empty strain *E. coli* AAEC191A, strain AAEC191A pHis-GFP that expresses GFP from pHis plasmid upon IPTG induction and strain AAEC191A rpsm-GFP carrying a plasmid to express GFP from the constitutive rpsm promoter. Per strain, bacteria from 20 fields of view (each 200x200 μm) were analyzed. Mean and standard deviation are shown and a two-independent sample two-sided t-test (α = 0.05) was performed. For the *E. coli* strains tested, the number of adherent bacteria was not significantly different (n.s.) with and without GFP expression. Population variances were not significantly different as tested by a two-sided F-test (α = 0.05). **(b)** Growth curves of *E. coli* K-12 derivative strains with and without plasmids for GFP expression. Turbidity of bacterial suspensions in 96 well plates was measured at 600 nm. Mean and standard deviation of a triplicate measurement are shown. **(c**,**d)** The fraction of adherent, GFP-fluorescent *E. coli* was analyzed. The empty strain (AAEC191A, n = 2468) was not fluorescent. GFP expression from both inducible and constitutive promoters yielded similar fractions of fluorescent *E. coli* (87.3% and 87.2%, respectively). A total of 2852 bacteria carrying the inducible *gfp* gene (pHis-GFP) and a total of 2036 bacteria carrying the constitutive *gfp* gene (rpsm-GFP) were analyzed. Shown are means and standard deviations. A two-independent sample two-sided t-test (α = 0.05) was performed and the fraction of fluorescent bacteria was not significantly different (n.s.) for the different GFP expressing *E. coli* strains. Population variances were not significantly different as tested by a two-sided F-test (α = 0.05).

For viability and growth kinetic studies of surface-adherent bacteria, *E. coli* that unspecifically adhered to bare glass surfaces were incubated in M9 growth medium that contained either 3 μM PI or a mixture of 6 μM SYTO® 9 and 30 μM PI (Figure 3a). For SYTO® 9 containing medium, *E. coli* replication and surface colonization was completely blocked, as determined by time lapse video microscopy (Figure 3a, Additional file 1: Figure S1). In addition to inhibiting bacterial growth, the viability of surface-adhering *E. coli* (AAEC191A), decreased for incubation times longer than 1.5 hours, as detected by two-channel fluorescence microscopy. In contrast, no decrease of viability or impaired growth was observed for endogenously eGFP expressing *E. coli* (AAEC191A pHis-GFP) counterstained with 3 μM PI (Figure 3a), which is in agreement with the results from the batch culture experiments (Figure 2). In controls we confirmed that GFP expression itself did not perturb *E. coli* adhesion and growth (Figure 4a,b). Furthermore, the fraction of GFP fluorescent *E. coli* (87%) was not significantly different (α = 0.05) for eGFP expression from the IPTG inducible pHis plasmid under the control of lac promoter (AAEC191A pHis-GFP) and under the control of a constitutive rpsm promoter (AAEC191A rpsm-GFP) (Figure 4c,d).

To evaluate whether the eGFP/3 μM PI assay is suited for *in situ* monitoring of bacterial viability and growth on a bioactive model substrate, eGFP-expressing *E. coli* (AAEC191A pHis-GFP) were incubated on antimicrobial dimethyloctadecyl[3-(trimethoxysilyl)propyl]ammonium chloride (DMOAC) coated glass surfaces [23, 27] (Figure 3b). Homogeneous DMOAC coating with a dry adlayer thickness of 2.2 nm was confirmed by variable-angle spectroscopic ellipsometry. To allow for a direct contact of the bacterial membrane with the surface-immobilized membrane-active DMOAC molecules, non-fimbriated eGFP expressing *E. coli* (AAEC191A pHis-GFP) were used [4]. As detected by 3 μM PI staining, all adherent bacteria on the DMOAC surface were killed within 30 minutes of surface incubation and no measurable bacterial growth occurred (Figure 3b, Additional file 1: Figure S2, Additional file 2: Movie S1).

### Pre-incubation of bioactive DMOAC surfaces with serum proteins completely blocked the antimicrobial activity and restored bacterial growth on the surface

To investigate if unspecific protein adsorption would interfere with the bactericidal activity of the DMOAC surfaces, the DMOAC surfaces were pre-incubated with fetal bovine serum (FBS) prior to bacterial incubation. Preconditioning of the antimicrobial surface with serum provides a model system for the rapid protein adsorption on biomaterial surfaces upon contact with host body fluids, notably blood, that can significantly impact the specific and unspecific binding of bacteria to the engineered material [10, 28]. Serum protein adsorption increased the dry adlayer thickness from 2.2 nm for pure DMOAC surfaces to 4.5 nm, as measured by ellipsometry. Using the optimized *in situ* eGFP/PI assay, we found that serum pre-incubation not only delayed but completely eliminated the bactericidal effect of the DMOAC surfaces on adherent *E. coli* (Figure 3b, Additional file 3: Movie S2). The bacteria survived and were able to divide on the protein-coated DMOAC surfaces. Division times of the surface-attached *E. coli* were comparable to those on bare control glass surfaces without bactericidal activity (Figure 3a).

## Discussion

A fluorescence based assay is introduced here that is well suited for the *in situ* monitoring of the viability and growth kinetics of surface-adhering and suspended bacteria. While we used *E. coli* as model organism, this assay should be applicable to other bacterial species as well if (i) the commonly used live DNA stains, such as SYTO^®^ 9, are replaced by endogenous eGFP expression and (ii) if the concentrations of propidium iodide (PI) needed to detect dead bacteria is reduced to non-toxic levels (3 μM for *E. coli*). While most available viability assays are restricted to suspended bacteria (i.e. CFU assay) or optical endpoint determinations (i.e. SYTO^®^ 9/PI LIVE/DEAD *Bac*Light™ viability kit, CTC assays), we show by fluorescence time-lapse microscopy and turbidity measurements, that reducing the PI concentration to 3 μM readily stained dead *E. coli* on bioactive DMOAC surfaces without disturbing bacterial growth (Figure 2, 3).

Our assay allows viability monitoring of single bacteria and emerging bacterial colonies. We should note though that the assay is not directly transferable to the study of mature biofilms without additional calibrations since the metabolic and genetic profile might change during biofilm mode of growth [29–31], and the synthesis of extracellular polymeric substances (EPS) [32, 33] might influence the bacterial GFP expression as well as the passive diffusion of PI through the biofilm matrix. Furthermore, detection of single bacteria within a dense three-dimensional biofilm matrix by epifluorescence microscopy might be challenging. However, since bacterial surface colonization starts with the adhesion of individual bacteria, the presented assay provides a versatile new tool for high spatial and temporal evaluation of bacterial viability on engineered surface coatings. The assay thus adds to the previously reported eGFP/PI flow cytometry assay that was limited to viability determination of suspended bacteria [21] and to the eGFP/PI endpoint viability study of groundwater *E. coli*[34].

Evaluating bacterial viability on the test surface omits the extraction of the adherent bacteria as required for solution based assays, e.g. CFU counts. Therefore, testing the bacterial viability on the substrate might increase the reliability of the assay since extraction is commonly achieved by ultrasonication or harsh washing procedures, both of which can harm bacteria. Furthermore, all CFU assays require prolonged incubation times for colony growth and are thus not applicable for real-time viability monitoring. As an alternative to extraction, an agar sandwich assay has been suggested to determine the viability of surface-adherent bacteria [35]. This method, however, is prone to errors, since each transferred bacterium will grow into a colony that in turn might overlap with colonies nearby. As an alternative direct optical viability assay of surface-adherent bacteria, the respiratory potential of bacteria can be monitored using 5-cyano-2,3-ditolyl tetrazolium chloride (CTC) [17]. The drawback however is that the CTC stain cannot be used for real-time monitoring since CTC disrupts the respiratory chain and is toxic to bacteria. This makes the CTC assay only suitable for end point determinations. We compared the performance of our eGFP/PI assay to the well-established SYTO^®^ 9/PI endpoint dual staining assay and found identical detection efficiencies of dead *E. coli* (Additional file 1: Figure S2). The SYTO^®^ 9/PI assay itself has been extensively compared to the above-mentioned viability tests [17, 19, 21, 26, 36] and showed comparative results to the solution based CFU assay as well as to other microscopy based endpoint viability protocols including the CTC assay. The added advantage of our assay is the ability to monitor the viability of adherent bacteria in real-time.

For each bacterial strain and species, one needs to optimize the PI concentrations to keep the bacteria viable, as done here for *Escherichia coli* (*E. coli*) K-12 MG1655. *E. coli* K-12 derivatives have been widely used as model strains in surface adhesion and biofilm studies [4, 31, 37–39]. We expressed eGFP from the pHis plasmid under the control of the IPTG inducible *tac* promoter to replace the growth inhibiting DNA stain SYTO^®^ 9 as live bacterial marker. To exclude negative effects of GFP expression as well as IPTG and antibiotic addition on bacterial viability and adhesion, we compared the adhesion properties and growth kinetics of the K-12 AAEC191A background strain [24] to a constitutive eGFP expressing strain (rpsm-GFP) and the IPTG inducible pHis-GFP strain (Figure 4a,b). No significant difference in adhesion and growth was observed. Furthermore, no significant difference in the fraction of GFP-expressing bacteria was found for the eGFP expression under the constitutive and inducible promoter used in this study (Figure 4c,d). However, even non-toxic gene products like GFP can have detrimental effects on bacteria when overexpressed [40], since overexpression of an introduced gene requires a lot of resources and thus might disorganize the bacterial metabolism. Therefore, the inducible IPTG-based expression system, as compared to constitutive expression systems, allows for a control of GFP expression and guarantees a balance between fluorescent and healthy bacteria (Figure 4c,d). Since eGFP is a very stable protein [41, 42] new GFP variants with reduced half-lives, e.g. GFP(LVA), have been suggested to study transient gene expression in bacteria [43]. The enzymatic degradation of unstable GFP(LVA) however, requires a metabolically active viable bacterium (Additional file 1: Figure S3). Thus, the use of GFP(LVA) variants does not improve our assay to limit false-positive detection of dead bacteria.

For live cell imaging, GFP expression in bacteria is used extensively and expression systems are available for many different bacterial species, including clinically relevant strains of *Salmonella*, *Streptococcus, Listeria monocytogenes*, *Pseudomonas aeruginosa, Staphylococcus aureus* and *Escherichia coli O157:H7*[41, 44–47]. Plasmid based gene expression is a well-established and long-used method in microbiology. Handling of plasmids is usually easy and versatile and genes, promoters or selection markers can quickly be exchanged and adapted to needs. Compared to plasmids, chromosomal insertions are more complicated and cannot be adapted as easily. Depending on the insertion method used, the gene of interest is inserted in the chromosome at a random location and might disrupt an important chromosomal gene. As for plasmids, selection markers like antibiotic resistances are commonly used for chromosomal insertions, too [48]. Furthermore, chromosomal insertions usually result in the insertion of a single copy of the gene into the chromosome. The pHis plasmid used in this study carries a ColE1-like replicator and occurs at nearly 20 copies per bacterium [49], each providing the gene of interest. This results in very stable and usually higher expression levels of the gene of interest (here eGFP) than with chromosomal insertions. Plasmids with ColE1-like promoters are stably inherited even without the presence of the corresponding selection agent [50, 51], long-term experiments with GFP expression from a plasmid rather than from a chromosomal insertion are feasible for more than a few hours. For other bacterial species and expression systems, the IPTG level and the concentration of the antibiotic selection marker should be re-evaluated to assure a stable GFP expression without disturbance of bacterial growth.

Finally, we applied our viability assay to highlight the impact of protein adsorption on the antimicrobial activity of engineered DMOAC surfaces. Upon incubation of the DMOAC surfaces with protein-rich fetal bovine serum, bacterial growth on the otherwise bactericidal surface was possible, indicating that the protein layer on top blocked the bioactive quaternary ammonium groups of the DMOAC coating (Figure 3b). The growth rate of the surface-attached *E. coli* on the control glass and serum-coated DMOAC substrates were identical, illustrating that the design rules for antimicrobial coatings primarily have to be tuned to prevent both, bacterial and protein adsorption since additional bioactive modifications can be lost when the biomaterial gets in contact with protein-rich (host) fluids.

## Conclusions

In conclusion, we show that the eGFP/PI assay is suited to study the antimicrobial properties of (bio-) material surface coatings under physiological conditions in real time and with single-bacterium sensitivity. This was so far not possible with the widely used solution based assays (i.e. CFU) or endpoint dual staining protocols (i.e. LIVE/DEAD *Bac*Light™ viability kit, CTC). Possible applications for the assay include studies of bacterial fitness and pathogenicity on biomaterial surfaces using live cell imaging of bacteria as additional readout. While we calibrated and illustrated the advantages of the assay for *E. coli*, other PI concentrations might have to be employed to optimize the kinetic viability monitoring of other bacterial species. While conventional bacteria viability assays allow for fast endpoint checks without requiring genetic modifications, the eGFP/PI assay presented here constitutes a viability test procedure that requires only one sample and its replicates per time series and is particularly suited for kinetic studies.

## Electronic supplementary material

Additional file 1: Figure S1: Protein coating reconstitutes bacterial growth on bioactive DMOAC surfaces as measured by an increase in bacterial surface coverage. The growth kinetics of 5–15 surface attached bacteria were analyzed and averaged for each condition. Error bars represent the standard deviation. **Figure S2**: The eGFP/PI and the SYTO^®^ 9/PI dual staining assays yield identical detection efficiencies of *E. coli* viability on bioactive DMOAC surfaces with fast bacteria deactivation kinetics (complete bacterial killing within 1 h incubation). **Figure S3**: Enzymatic degradation of GFP variants with different stability. All *E. coli* strains express GFP from plasmid pHis under control of the inducible *tac* promoter. At time point 0 h GFP expression was stopped by removing the IPTG inducer. *E. coli* expressing the stable eGFP variant showed the highest fluorescence intensity and nearly no degradation within 4.5 h. Strains that expressed the unstable GFP(LVA) variant exhibited an inherent lower fluorescence intensity from the start, as the unstable GFP(LVA) was constantly being degraded by innate *E. coli* proteases. When GFP(LVA) expression was stopped by IPTG removal and the culture is maintained at 37°C, the GFP fluorescence decreased rapidly, indicating that the GFP(LVA) is degraded enzymatically. If the culture was kept at 0°C after IPTG removal, no degradation of the GFP(LVA) was observed. All measurements were performed in M9 minimal medium. 1 ml samples were drawn at each time point and measured with a Perkin Elmer spectrophotometer. OD_600_ of all cultures at 0 h was set to 1. (PDF 361 KB)

Additional file 2: Movie S1: DMOAC-coated bioactive surfaces show fast killing kinetics of surface-attached *E. coli* bacteria. Time-lapse data of fluorescent images of surface-bound *E. coli* bacteria (green: eGFP signal, red: PI signal) is shown. (MOV 2 MB)

Additional file 3: Movie S2: Incubation of serum with DMOAC-coated bioactive surfaces eliminates bioactive effect of DMOAC surfaces and rescues *E. coli* viability and growth. Time-lapse data of fluorescent images of surface-bound *E. coli* bacteria (green: eGFP signal, red: PI signal) is shown. (MOV 253 KB)
