# Supplementary material for: Fluorescence-based in situ assay to probe the viability and growth kinetics of surface-adhering and suspended recombinant bacteria
Source: Biointerphases. 2013 Aug 21;8(1):22. doi: 10.1186/1559-4106-8-22 (PMC4243816; doi:10.1186/1559-4106-8-22)
Supplement: Supplementary file 1 — Additional file 1: Figure S1: Protein coating reconstitutes bacterial growth on bioactive DMOAC surfaces as measured by an increase in bacterial surface coverage. The growth kinetics of 5–15 surface attached bacteria were analyzed and averaged for each condition. Error bars represent the standard deviation. Figure S2: The eGFP/PI and the SYTO® 9/PI dual staining assays yield identical detection efficiencies of E. coli viability on bioactive DMOAC surfaces with fast bacteria deactivation kinetics (complete bacterial killing within 1 h incubation). Figure S3: Enzymatic degradation of GFP variants with different stability. All E. coli strains express GFP from plasmid pHis under control of the inducible tac promoter. At time point 0 h GFP expression was stopped by removing the IPTG inducer. E. coli expressing the stable eGFP variant showed the highest fluorescence intensity and nearly no degradation within 4.5 h. Strains that expressed the unstable GFP(LVA) variant exhibited an inherent lower fluorescence intensity from the start, as the unstable GFP(LVA) was constantly being degraded by innate E. coli proteases. When GFP(LVA) expression was stopped by IPTG removal and the culture is maintained at 37°C, the GFP fluorescence decreased rapidly, indicating that the GFP(LVA) is degraded enzymatically. If the culture was kept at 0°C after IPTG removal, no degradation of the GFP(LVA) was observed. All measurements were performed in M9 minimal medium. 1 ml samples were drawn at each time point and measured with a Perkin Elmer spectrophotometer. OD600 of all cultures at 0 h was set to 1. (PDF 361 KB) [file BJIOBN-000008-000022_1-s001.pdf]

Supplementary information

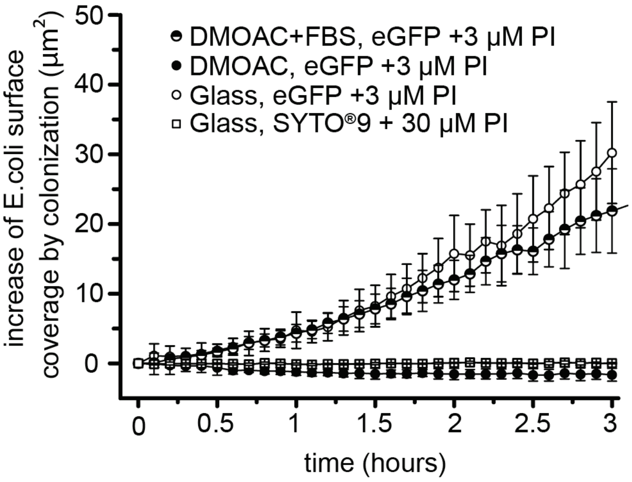

Figure S1. Protein coating reconstitutes bacterial growth on bioactive DMOAC surfaces as measured by an increase in bacterial surface coverage. The growth kinetics of 5-15 surface attached bacteria were analyzed and averaged for each condition. Error bars represent the standard deviation.

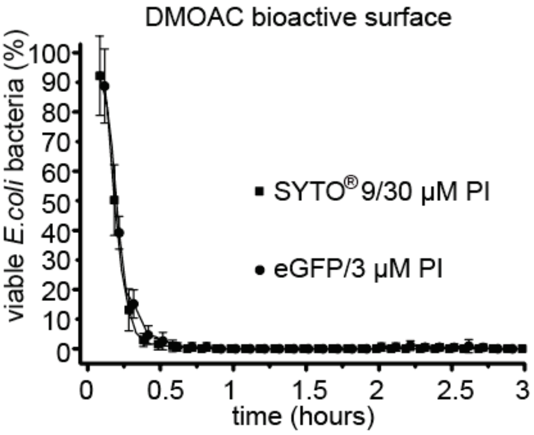

Figure S2. The eGFP/PI and the SYTO® 9/PI dual staining assays yield identical detection efficiencies of *E. coli* viability on bioactive DMOAC surfaces with fast bacteria deactivation kinetics (complete bacterial killing within 1 h incubation).

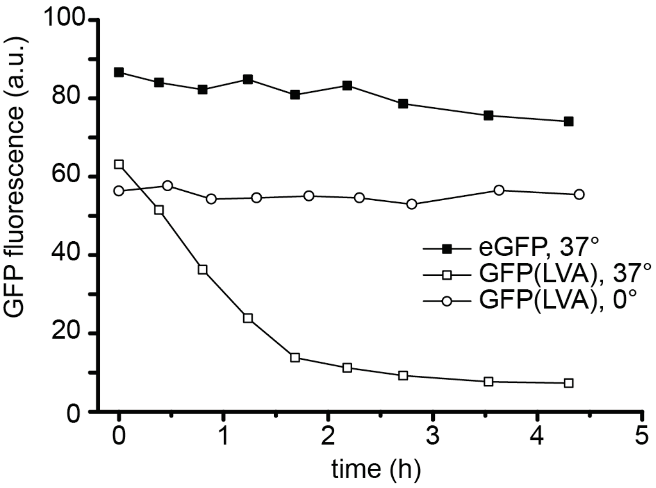

Figure S3. Enzymatic degradation of GFP variants with different stability. All *E. coli* strains express GFP from plasmid pHis under control of the inducible tac promoter. At time point 0 h GFP expression was stopped by removing the IPTG inducer. *E. coli* expressing the stable eGFP variant showed the highest fluorescence intensity and nearly no degradation within 4.5 h. Strains that expressed the unstable GFP(LVA) variant exhibited an inherent lower fluorescence intensity from the start, as the unstable GFP(LVA) was constantly being degraded by innate *E. coli* proteases. When GFP(LVA) expression was stopped by IPTG removal and the culture is maintained at 37 °C, the GFP fluorescence decreased rapidly, indicating that the GFP(LVA) is degraded enzymatically. If the culture was kept at 0 °C after IPTG removal, no degradation of the GFP(LVA) was observed. All measurements were performed in M9 minimal medium. 1 ml samples were drawn at each time point and measured with a Perkin Elmer spectrophotometer. OD_{600} of all cultures at 0 h was set to 1.

Supplemental Movie S1. DMOAC-coated bioactive surfaces show fast killing kinetics of surface-attached *E. coli* bacteria. Time-lapse data of fluorescent images of surface-bound *E. coli* bacteria (green: eGFP signal, red: PI signal) is shown.

Supplemental Movie S2. Incubation of serum with DMOAC-coated bioactive surfaces eliminates bioactive effect of DMOAC surfaces and rescues *E. coli* viability and growth. Time-lapse data of fluorescent images of surface-bound *E. coli* bacteria (green: eGFP signal, red: PI signal) is shown.

Image analysis codes in MATLAB®

1) Main file for data sorting

```
%generates the conditions for selection and renaming of images and further
%processing
%written for data of mutliple stage positions that have to be sorted into
%distinct folders for further analysis.
%written by Philipp Miermeister, contact philipp.miermeister@ipa.fhg.de or
%ima.avalos@hest.ethz.ch for questions

basePath = '…';
folder_Images = 'Images\';
folder_dia = 'Dia\';
folder_green = 'FITC\';
folder_red = 'Red\';
fileName_Dia_Pos = '130424_w1Dia Illumination_s'; %case sensitive, has to be adjusted to the employed channel (Dia, Dia DIC, ...)
fileName_fitc_Pos = '130424_w2FITC BP_s';
fileName_red_Pos = '130424_w3Rhodamine_s';


numI = 67;         % Set number of images assembled in image stack =number of timepoints
numPos = 3;        % Set number of positions acquired in Metamorph MultiDim. Acquisition
%----------------------------------------------------------------------
%% create 'images' directory and add it to matlab path
[status, message, messageid] = mkdir ([basePath, folder_Images]);
addpath([basePath, folder_Images]);
%% create position subfolders in 'images' directory
for i=1:numPos

    mkdir([basePath, folder_Images, 's', num2str(i, '%0.2d')]);      %creates as many folders as position numbers specified
    addpath([basePath, folder_Images, 's', num2str(i, '%0.2d')]);   %adds position folders to matlab path

    mkdir([basePath, folder_Images, 's', num2str(i, '%0.2d'), '\', folder_dia]);
    addpath([basePath, folder_Images, 's', num2str(i, '%0.2d'), '\', folder_dia]);

    mkdir([basePath, folder_Images, 's', num2str(i, '%0.2d'), '\', folder_green]);
    addpath([basePath, folder_Images, 's', num2str(i, '%0.2d'), '\', folder_green]);

    mkdir([basePath, folder_Images, 's', num2str(i, '%0.2d'), '\', folder_red]);
    addpath([basePath, folder_Images, 's', num2str(i, '%0.2d'), '\', folder_red]);
end
```

```
%%

for i=1:numPos
    %20121212_beads_lps_fim_rawt001xy01c1
    sourceFile=[basePath, fileName_Dia_Pos, num2str(i, '%0.1d'), '*', '.tif'];
    destFile=[basePath, folder_Images, 's', num2str(i, '%0.2d')];
    copyfile (sourceFile, destFile);

    sourceFile=[basePath, fileName_fitc_Pos, num2str(i, '%0.1d'), '*', '.tif'];
    destFile=[basePath, folder_Images, 's', num2str(i, '%0.2d')];
    copyfile (sourceFile, destFile);

    sourceFile=[basePath, fileName_red_Pos, num2str(i, '%0.1d'), '*', '.tif'];
    destFile=[basePath, folder_Images, 's', num2str(i, '%0.2d')];
    copyfile (sourceFile, destFile);
end
```

2) Main file for image import

```
%% Import script for microscopy images
% Take care that you change to the correct current folder!!
% Raw data folder contains all 3 types of images

% One can increase Java memory by setting the Java Heap Memory
% This can be used to avoid Java heap space errors!
% File > Preferences > General > Java Heap Memory.
% http://bigwww.epfl.ch/sage/soft/mij/

%written by Philipp Miermeister, contact philipp.miermeister@ipa.fhg.de or
%ima.avalos@hest.ethz.ch for questions
%%
clear all;

%% User specific configurations
%-------------------------------------------------------------------------
basePath = 'C:\Users\aima\Desktop\viabilityRemakeForVideo\Images\s03\';
fileName_Dia = '130424_w1Dia Illumination_s3_t';      %change date and position number
fileName_fitc = '130424_w2FITC BP_s3_t';
fileName_red = '130424_w3Rhodamine_s3_t';

numI = 59;          % Set number of images assembled in image sequence
stepSize = 1;       % Adjust according to exposure frequency
stepSize_Dia = stepSize;    % Adjust according to microscopy error during Dia channel acquisition, if no error occurred, this variable has the same value as the stepSize
DiaMult = 1;


%-------------------------------------------------------------------------

folder_Images = 'images\';
folder_Output_Dia = 'Output\dia\';
folder_Output_Fitc = 'Output\fitc\';
folder_Output_Red = 'Output\red\';

folder_regInput_Dia = 'registrationInput\dia\';
folder_regInput_Fitc = 'registrationInput\fitc\';
folder_regInput_Red = 'registrationInput\red\';
folder_transforms = 'registrationInput\transforms\';

folder_cropped = 'cropped\';
folder_cropped_Dia = 'cropped\dia\';
folder_cropped_Fitc = 'cropped\fitc\';
folder_cropped_Red = 'cropped\red\';

%% Create folders if non existent
```

```
[status,message,messageid] = mkdir([basePath, folder_Images]);
[status,message,messageid] = mkdir([basePath, folder_Output_Dia]);
[status,message,messageid] = mkdir([basePath, folder_Output_Fitc]);
[status,message,messageid] = mkdir([basePath, folder_Output_Red]);
[status,message,messageid] = mkdir([basePath, folder_regInput_Dia]);
[status,message,messageid] = mkdir([basePath, folder_regInput_Fitc]);
[status,message,messageid] = mkdir([basePath, folder_regInput_Red]);
[status,message,messageid] = mkdir([basePath, folder_transforms]);
[status,message,messageid] = mkdir([basePath, folder_cropped]);
[status,message,messageid] = mkdir([basePath, folder_cropped_Dia]);
[status,message,messageid] = mkdir([basePath, folder_cropped_Fitc]);
[status,message,messageid] = mkdir([basePath, folder_cropped_Red]);

%% add folders to matlab path

addpath([basePath, folder_Images]);
addpath([basePath, 'Output']);
addpath([basePath, 'registrationInput']);
addpath([basePath, folder_Output_Dia]);
addpath([basePath, folder_Output_Fitc]);
addpath([basePath, folder_Output_Red]);
addpath([basePath, folder_regInput_Dia]);
addpath([basePath, folder_regInput_Fitc]);
addpath([basePath, folder_regInput_Red]);
addpath([basePath, folder_transforms]);
addpath([basePath, folder_cropped]);
addpath([basePath, folder_cropped_Dia]);
addpath([basePath, folder_cropped_Fitc]);
addpath([basePath, folder_cropped_Red]);
%%
clear folders
delete([basePath, folder_Output_Dia, '*']);
delete([basePath, folder_Output_Fitc,'*']);
delete([basePath, folder_Output_Red, '*']);
delete([basePath, folder_regInput_Dia, '*']);
delete([basePath, folder_regInput_Fitc, '*']);
delete([basePath, folder_regInput_Red, '*']);
delete([basePath, folder_transforms, '*']);
delete([basePath, folder_cropped, '*']);



%%
sizeX_org = 1000;
sizeY_org = 1000;

%%
%I_org = uint16(zeros(sizeX_org, sizeY_org, numI));

% Rename and duplicate Dia images into registration folders (duplicate only if
% not all images were recorded correctly)
%-------------------------------------------------------------------------

i_out = 1;

for i_in = 1:stepSize_Dia:numI
    nameStr_Read = [basePath, fileName_Dia, num2str(i_in, '%0.1d'), '.tif'];
    I = imread(nameStr_Read);

    % copy images stepSize_Dia times
    repeats = DiaMult;
    for j = 1:1:repeats
        if i_out <= numI
            nameStr_Write = [basePath, folder_regInput_Dia, fileName_Dia, num2str(i_out, '%0.3d'), '.tif'];
            imwrite(I,nameStr_Write);
            i_out = i_out + stepSize_Dia / repeats;
        else
```

```
            % skip copying if last image has been reached
        end
    end
end

fprintf('Yeah I finished copying the *$#@! Dia images!\n');

% Rename fitc and red image files and copy to registrationInput folder
%--------------------------------------------------------------------------
for i = 1:stepSize:numI
    nameStr_Read = [basePath, fileName_fitc, num2str(i, '%0.1d'), '.tif'];
    nameStr_Write = [basePath, folder_regInput_Fitc, fileName_fitc, num2str(i, '%0.3d'), '.tif'];
    I = imread(nameStr_Read);
    imwrite(I,nameStr_Write);

    nameStr_Read = [basePath, fileName_red, num2str(i, '%0.1d'), '.tif'];
    nameStr_Write = [basePath, folder_regInput_Red, fileName_red, num2str(i, '%0.3d'), '.tif'];
    I = imread(nameStr_Read);
    imwrite(I,nameStr_Write);


%    MIJ.createColor(['mpic',num2str(i, '%0.2d')], Irgb8(:,:,:,i), 1);
%    I_org_i = imread([folder, stackFileName],i); % load ith image of stack
%    imwrite(I_org_i,

end

%--------------------------------------------------------------------------
fprintf('Now I am tired, but I did my work!\n');
fprintf('All images were renamed and stored in \n registrationInput dia, fitc and red. \n\n');
%fprintf('I do not have enough memory, please use ImageJ\Plugins\Registration\Register Virtual Stack Slices and ImageJ\Plugins\Transform\Transform Virtual Stack Slices to register the slices.\n');
%%
%register Dia images and store transform files for registration of fluorescence
%images, Miji pligin of Fiji required! set scripts folder of Fiji to Matlab
%path.
% folder_regInput_Dia(1:end-1) use all elements but the last one




% fprintf('I will start the registration now, please be patient and dont bother me\n');
%
% macroParameters = ['source=[', basePath, folder_regInput_Dia(1:end-1), '] ' ...
%               'output=[', basePath, folder_Output_Dia(1:end-1), '] ' ...
%               'feature=Translation ' ...
%               'save'...
%               ];
%
% MIJ.run('Register Virtual Stack Slices', macroParameters);
%
% fprintf('All Dia images were stabilized. \n');
%
% %%
% % register green and red channel images using Dia images
% macroParameters = ['source=[', basePath, folder_regInput_Fitc(1:end-1), '] ' ...
%               'output=[', basePath, folder_Output_Fitc(1:end-1), '] ' ...
%               'transforms=[', basePath, folder_transforms(1:end-1), '] ' ...
%               'interpolate'];
% MIJ.run('Transform Virtual Stack Slices', macroParameters);
% fprintf('Green channels were stabilized. \n');
% % register red channel images using Dia images
%
% macroParameters = ['source=[', basePath, folder_regInput_Red(1:end-1), '] ' ...
%               'output=[', basePath, folder_Output_Red(1:end-1), '] ' ...
%               'transforms=[', basePath, folder_transforms(1:end-1), '] ' ...
```

```
%               'interpolate'];
% MIJ.run('Transform Virtual Stack Slices', macroParameters);
% fprintf('Red channels were stabilized. \n');
%
%
% % Crop images
% %%
% fprintf('Start cropping images.\n');
% i = 1;
% % Initialize I_crop with first picture
% I_crop = imread([basePath, folder_Output_Dia ,fileName_Dia ,num2str(i, '%0.3d') '.tif']);
%
% % Combine images logically
% for i = 1+stepSize:stepSize:numI
%     I = imread([basePath, folder_Output_Dia ,fileName_Dia ,num2str(i, '%0.3d') '.tif']);
%     I_crop = and(I_crop, I);
% end
%
% %imshow(I_crop);
%
%
% props = regionprops(I_crop, 'BoundingBox');
% ul_x = round(props.BoundingBox(1))+1;
% ul_y = round(props.BoundingBox(2))+1;
% size_xy = min(props.BoundingBox(3:4))-1;
%
% % make size_xy even
% if (mod(size_xy,2) ~= 0) % if odd
%     size_xy = size_xy -1;
% end
%
% size_xy = size_xy - 1; % reduce size_xy due to the sum in the following matrix indexing operation
% % Crop images and save
% for i = 1:stepSize:numI
%     % Dia channel
%     I = imread([basePath, folder_Output_Dia ,fileName_Dia ,num2str(i, '%0.3d') '.tif']);
%     I = I(ul_y:ul_y+size_xy, ul_x:ul_x+size_xy);
%     imwrite(I,[basePath, folder_cropped_Dia, fileName_Dia ,num2str(i, '%0.3d') '.tif']);
%
%     % Fitc channel
%     I = imread([basePath, folder_Output_Fitc ,fileName_fitc ,num2str(i, '%0.3d') '.tif']);
%     I = I(ul_y:ul_y+size_xy, ul_x:ul_x+size_xy);
%     imwrite(I,[basePath, folder_cropped_Fitc, fileName_fitc ,num2str(i, '%0.3d') '.tif']);
%
%     % red channel
%     I = imread([basePath, folder_Output_Red ,fileName_red ,num2str(i, '%0.3d') '.tif']);
%     I = I(ul_y:ul_y+size_xy, ul_x:ul_x+size_xy);
%     imwrite(I,[basePath, folder_cropped_Red, fileName_red ,num2str(i, '%0.3d') '.tif']);
%
% end
% fprintf('Finished cropping images. continue to next script Q1CountBacteria\n');
% %imshow(I);
%
% %%
%
% %%
% % run("Transform Virtual Stack Slices",
% % "source=[C:\Users\Philipp\Documents\MATLAB\Viability 2011_12_30
% %     \122211CoatingD\s9-no journal every image acquired\registrationInput\fitc]
% % output=[C:\Users\Philipp\Documents\MATLAB\Viability 2011_12_30\122211CoatingD
% %     \s9-no journal every image acquired\Output\fitc]
% % transforms=[C:\Users\Philipp\Documents\MATLAB\Viability 2011_12_30\122211CoatingD\
% %     s9-no journal every image acquired\registrationInput\transforms] interpolate");
%
% % run("Register Virtual Stack Slices",
% % "source=[C:\Users\Philipp\Documents\MATLAB\Viability 2011_12_30\122211CoatingD\s9-no
% %     journal every image acquired\registrationInput\Dia]
```

```
% % output=[C:\Users\Philipp\Documents\MATLAB\Viability 2011_12_30\122211CoatingD\s9-no
% %     journal every image acquired\Output\dia] feature=Translation registration=[Translation
% %     -- no deformation                    ] save");
%
%
%
% % imshow(I_org(:,:,1));
```

3) Main file for image stabilization

```
%% Import script for microscopy images
% Take care that you change to the correct current folder!!
% Raw data folder contains all 3 types of images

% One can increase Java memory by setting the Java Heap Memory
% This can be used to avoid Java heap space errors!
% File > Preferences > General > Java Heap Memory.
% http://bigwww.epfl.ch/sage/soft/mij/


%written by Philipp Miermeister, contact philipp.miermeister@ipa.fhg.de or
%ima.avalos@hest.ethz.ch for questions
%%
clear all;

%% User specific configurations
%--------------------------------------------------------------------------
basePath = 'C:\Users\aima\Desktop\viabilityRemakeForVideo\Images\s03\';
fileName_Dia = '130424_w1Dia Illumination_s3_t';      %change date and position number
fileName_fitc = '130424_w2FITC BP_s3_t';
fileName_red = '130424_w3Rhodamine_s3_t';

numI = 59;          % Set number of images assembled in image sequence
stepSize = 1;        % Adjust according to exposure frequency
stepSize_Dia = stepSize;    % Adjust according to microscopy error during Dia channel acquisition, if no
error occurred, this variable has the same value as the stepSize
DiaMult = 1;


%--------------------------------------------------------------------------

folder_Images = 'images\';
folder_Output_Dia = 'Output\dia\';
folder_Output_Fitc = 'Output\fitc\';
folder_Output_Red = 'Output\red\';

folder_regInput_Dia = 'registrationInput\dia\';
folder_regInput_Fitc = 'registrationInput\fitc\';
folder_regInput_Red = 'registrationInput\red\';
folder_transforms = 'registrationInput\transforms\';

folder_cropped = 'cropped\';
folder_cropped_Dia = 'cropped\dia\';
folder_cropped_Fitc = 'cropped\fitc\';
folder_cropped_Red = 'cropped\red\';

%% Create folders if non existent

[status,message,messageid] = mkdir([basePath, folder_Images]);
[status,message,messageid] = mkdir([basePath, folder_Output_Dia]);
[status,message,messageid] = mkdir([basePath, folder_Output_Fitc]);
[status,message,messageid] = mkdir([basePath, folder_Output_Red]);
[status,message,messageid] = mkdir([basePath, folder_regInput_Dia]);
[status,message,messageid] = mkdir([basePath, folder_regInput_Fitc]);
[status,message,messageid] = mkdir([basePath, folder_regInput_Red]);
[status,message,messageid] = mkdir([basePath, folder_transforms]);
```

```
[status,message,messageid] = mkdir([basePath, folder_cropped]);
[status,message,messageid] = mkdir([basePath, folder_cropped_Dia]);
[status,message,messageid] = mkdir([basePath, folder_cropped_Fitc]);
[status,message,messageid] = mkdir([basePath, folder_cropped_Red]);

%% add folders to matlab path

addpath([basePath, folder_Images]);
addpath([basePath, 'Output']);
addpath([basePath, 'registrationInput']);
addpath([basePath, folder_Output_Dia]);
addpath([basePath, folder_Output_Fitc]);
addpath([basePath, folder_Output_Red]);
addpath([basePath, folder_regInput_Dia]);
addpath([basePath, folder_regInput_Fitc]);
addpath([basePath, folder_regInput_Red]);
addpath([basePath, folder_transforms]);
addpath([basePath, folder_cropped]);
addpath([basePath, folder_cropped_Dia]);
addpath([basePath, folder_cropped_Fitc]);
addpath([basePath, folder_cropped_Red]);
%%
% clear folders
% delete([basePath, folder_Output_Dia, '*']);
% delete([basePath, folder_Output_Fitc,'*']);
% delete([basePath, folder_Output_Red, '*']);
% delete([basePath, folder_regInput_Dia, '*']);
% delete([basePath, folder_regInput_Fitc, '*']);
% delete([basePath, folder_regInput_Red, '*']);
% delete([basePath, folder_transforms, '*']);
% delete([basePath, folder_cropped, '*']);
%


%%
sizeX_org = 1000;
sizeY_org = 1000;


%%
%register Dia images and store transform files for registration of fluorescence
%images, Miji pligin of Fiji required! set scripts folder of Fiji to Matlab
%path.
% folder_regInput_Dia(1:end-1) use all elements but the last one
fprintf('I will start the registration now, please be patient and dont bother me\n');

macroParameters = ['source=[', basePath, folder_regInput_Dia(1:end-1), '] ' ...
        'output=[', basePath, folder_Output_Dia(1:end-1), '] ' ...
        'feature=Translation ' ...
        'save'...
        ];

MIJ.run('Register Virtual Stack Slices', macroParameters);

fprintf('All Dia images were stabilized. \n');

%%
% register green and red channel images using Dia images
macroParameters = ['source=[', basePath, folder_regInput_Fitc(1:end-1), '] ' ...
        'output=[', basePath, folder_Output_Fitc(1:end-1), '] ' ...
        'transforms=[', basePath, folder_transforms(1:end-1), '] ' ...
        'interpolate'];
MIJ.run('Transform Virtual Stack Slices', macroParameters);
fprintf('Green channels were stabilized. \n');
% register red channel images using Dia images

macroParameters = ['source=[', basePath, folder_regInput_Red(1:end-1), '] ' ...
```

```
            'output=[', basePath, folder_Output_Red(1:end-1), '] ' ...
            'transforms=[', basePath, folder_transforms(1:end-1), '] ' ...
            'interpolate'];
MIJ.run('Transform Virtual Stack Slices', macroParameters);
fprintf('Red channels were stabilized. \n');


% Crop images
%%
fprintf('Start cropping images.\n');
i = 1;
% Initialize I_crop with first picture
I_crop = imread([basePath, folder_Output_Dia ,fileName_Dia ,num2str(i, '%0.3d') '.tif']);

% Combine images logically
for i = 1+stepSize:stepSize:numI
   I = imread([basePath, folder_Output_Dia ,fileName_Dia ,num2str(i, '%0.3d') '.tif']);
   I_crop = and(I_crop, I);
end

%imshow(I_crop);


props = regionprops(I_crop, 'BoundingBox');
ul_x = round(props.BoundingBox(1))+1;
ul_y = round(props.BoundingBox(2))+1;
size_xy = min(props.BoundingBox(3:4))-1;

% make size_xy even
if (mod(size_xy,2) ~= 0) % if odd
   size_xy = size_xy -1;
end

size_xy = size_xy - 1; % reduce size_xy due to the sum in the following matrix indexing operation
% Crop images and save
for i = 1:stepSize:numI
   % Dia channel
   I = imread([basePath, folder_Output_Dia ,fileName_Dia ,num2str(i, '%0.3d') '.tif']);
   I = I(ul_y:ul_y+size_xy, ul_x:ul_x+size_xy);
   imwrite(I,[basePath, folder_cropped_Dia, fileName_Dia ,num2str(i, '%0.3d') '.tif']);

   % Fitc channel
   I = imread([basePath, folder_Output_Fitc ,fileName_fitc ,num2str(i, '%0.3d') '.tif']);
   I = I(ul_y:ul_y+size_xy, ul_x:ul_x+size_xy);
   imwrite(I,[basePath, folder_cropped_Fitc, fileName_fitc ,num2str(i, '%0.3d') '.tif']);

   % red channel
   I = imread([basePath, folder_Output_Red ,fileName_red ,num2str(i, '%0.3d') '.tif']);
   I = I(ul_y:ul_y+size_xy, ul_x:ul_x+size_xy);
   imwrite(I,[basePath, folder_cropped_Red, fileName_red ,num2str(i, '%0.3d') '.tif']);

end
fprintf('Finished cropping images. continue to next script Q1CountBacteria\n');
%imshow(I);

%%

%%
% run("Transform Virtual Stack Slices",
% "source=[C:\Users\Philipp\Documents\MATLAB\Viability 2011_12_30
%    \122211CoatingD\s9-no journal every image acquired\registrationInput\fitc]
% output=[C:\Users\Philipp\Documents\MATLAB\Viability 2011_12_30\122211CoatingD
%    \s9-no journal every image acquired\Output\fitc]
% transforms=[C:\Users\Philipp\Documents\MATLAB\Viability 2011_12_30\122211CoatingD\
%    s9-no journal every image acquired\registrationInput\transforms] interpolate");

% run("Register Virtual Stack Slices",
```

```
% "source=[C:\Users\Philipp\Documents\MATLAB\Viability 2011_12_30\122211CoatingD\s9-no
%     journal every image acquired\registrationInput\Dia]
% output=[C:\Users\Philipp\Documents\MATLAB\Viability 2011_12_30\122211CoatingD\s9-no
%     journal every image acquired\Output\dia] feature=Translation registration=[Translation
%     -- no deformation                    ] save");



% imshow(I_org(:,:,1));
```

4) Main file for image segmentation

```
%written by Philipp Miermeister, contact philipp.miermeister@ipa.fhg.de or
%ima.avalos@hest.ethz.ch for questions


close all; % Close windows
clear all;
%clear all; % Clear workspace
clc; % Clear command window

runTime = tic;

%% User specific configurations
%--------------------------------------------------------------------------

fileStruct.basePath                                                                                                                        =
'R:\ExchangeServerAndBackups\BackupMathilda\ExperimentsOnDMOAC\021712AAEC_5%DMOAC_4
0x_300_300msexp\images\s04\';
fileStruct.fileName_Dia = '0217_w1Dia Illumination_s4_t';
fileStruct.fileName_fitc = '0217_w2FITC BP_s4_t';
fileStruct.fileName_red = '0217_w3Rhodamine_s4_t';
fileStruct.fileNameVar = '0217_s4_singlebacccis_0420';

numI = 55;              % Set number of images assembled in image stack
stepSize = 2;           % Adjust according to exposure frequency
negOffset = 0;
edgeFilterSize = 10;    % Distance from image border where bacteria will be removed
diskSize = 15;          % Disk size for background subtraction


% Adjusting images by user defined values
levelG = ones(numI,1) * 0.025;
levelR = ones(numI,1) * 0.1;
%%
%--------------------------------------------------------------------------
% create file structure
fileStruct.folder_Images = 'images\';
fileStruct.folder_Output_Dia = 'Output\dia\';
fileStruct.folder_Output_Fitc = 'Output\fitc\';
fileStruct.folder_Output_Red = 'Output\red\';

fileStruct.folder_regInput_Dia = 'registrationInput\dia\';
fileStruct.folder_regInput_Fitc = 'registrationInput\fitc\';
fileStruct.folder_regInput_Red = 'registrationInput\red\';

fileStruct.folder_transforms = 'registrationInput\transforms\';
fileStruct.folder_cropped = 'cropped\';
%--------------------------------------------------------------------------

fprintf('load images\n');
fprintf([num2str(toc(runTime), '%0.1f'), 's\n']);
[I_Dia, I_R, I_G] = loadImages(fileStruct, numI, stepSize);

fprintf('crop images\n');
[I_Dia, I_R, I_G] = cropImages(I_Dia, I_R, I_G, negOffset);
```

```
% Adjust each image based on the min/max value found in the time series
fprintf([num2str(toc(runTime), '%0.1f'), 's\n']);
fprintf('Adjust image histogram by scaling\n');
[I_Dia, I_R, I_G] = adjustImageHistogram(I_Dia, I_R, I_G, numI, stepSize);

fprintf([num2str(toc(runTime), '%0.1f'), 's\n']);
fprintf(['Compute background for R and G using disk element of size ', num2str(diskSize), '\n']);
[IR_BC1, IG_BC1] = removeBackground(I_R, I_G, numI, stepSize);

surf(double(IG_BC1(1:10:end,1:10:end,1))),zlim([0 20000]);
%implay(background_R1);
%%
% Convert greyscale to rgb
fprintf([num2str(toc(runTime), '%0.1f'), 's\n']);
fprintf('Convert greyscale to rgb\n');



%Thresholding and greyscale-to-bw conversion of the green images
minObjectSize = 40;

bwG = grey2bw(IG_BC1, levelG, minObjectSize, numI);
bwR = grey2bw(IR_BC1, levelR, minObjectSize, numI);
%%

%imshow(bwG(:,:,3));

%%
% Combine green bw and red bw images
fprintf([num2str(toc(runTime), '%0.1f'), 's\n']);
fprintf('combining images\n');
bwG_add = combine_bwImageSeries(bwG, 'or', numI);
bwR_add = combine_bwImageSeries(bwR, 'or', numI);

 %% Remove bacteria near image borders
 % use last label image of cumulated time series as reference for the border
 % removal operation
fprintf([num2str(toc(runTime), '%0.1f'), 's\n']);
fprintf('removing border elements\n');
L_G_add = label_bwImageSeries(bwG_add, numI);
L_ref =  L_G_add(:,:,end);
bwG = removeBorderElements(L_ref, bwG, edgeFilterSize, numI);
bwG_add= removeBorderElements(L_ref, bwG_add, edgeFilterSize, numI);
% The removal odf the red bacteria may cause artefacts int the
% visualization, because only parts of the bacteria are removed that are
% lying inside the green area
%bwR = removeBorderElements(L_ref, bwR, edgeFilterSize, numI);
%bwR_add = removeBorderElements(L_ref, bwR_add, edgeFilterSize, numI);
%%

%labeled images
fprintf([num2str(toc(runTime), '%0.1f'), 's\n']);
fprintf('labeling images\n');
L_G_noadd = label_bwImageSeries(bwG, numI);
L_G_add = label_bwImageSeries(bwG_add, numI);


%% compute living and dead cell list
CellRefLabelFrame = L_G_add(:,:,end);
cellCountG_noadd = computeCellList(CellRefLabelFrame, bwG, numI );
cellCountG_add = computeCellList(CellRefLabelFrame, bwG_add, numI );
%%
cellCountR_noadd = computeCellList(CellRefLabelFrame, bwR, numI );
cellCountR_add= computeCellList(CellRefLabelFrame, bwR_add, numI );

%% Create color images from greyscale images
fprintf([num2str(toc(runTime), '%0.1f'), 's\n']);
```

```
fprintf('creating color images\n');
intensityG = 2;
intensityR = 3;
%Irgb = combineImagesToRGB(I_Dia, IG_BC1, IR_BC1, bwG, bwR_add, intensityG, intensityR, numI);
%uncomment line above and comment line two lines below if black background of fluorescence signal wanted
I_Dia(:,:,:)=0;

Irgb = combineImagesToRGB(I_Dia, uint16(bwG_add)*10000, uint16(bwR_add)*10000, bwG_add, bwR_add, intensityG, intensityR, numI);
%comment upper line and uncomment line two lines above if Dia image
%background is wanted for fluorescence signal
%%
for i = 1:stepSize:numI
    %calculate loss of viability
    c_Loss_V = cellCountR_add./cellCountG_add*100;

    c_Viab = 100-c_Loss_V;

    %numG_addmax=max(cellCountG_add);
    %c_Loss_V_max=cellCountR_add./numG_addmax*100;
        %subplot(1,2,2), subimage(bwG);
    %imshow(bwG);

    % combine images into so far zerofilled rgb channels


    %imshow (Irgb(:,:,:,i));

    %calculate relative bleaching of green and red channel
    c_Bleaching_G = cellCountG_noadd./cellCountG_add*100;
    c_Bleaching_R = cellCountR_noadd./cellCountG_add*100;
end
fprintf([num2str(toc(runTime), '%0.1f'), 's\n']);
fprintf('478: Finished image analysis.\n');
%%
fprintf([num2str(toc(runTime), '%0.1f'), 's\n']);
fprintf('481: plotting viability data now.\n');

plot(1:stepSize:numI,cellCountR_add(1:stepSize:end), 'r');
hold on;
plot(1:stepSize:numI,cellCountG_add(1:stepSize:end), 'g');
hold on;
plot(1:stepSize:numI,cellCountR_noadd(1:stepSize:end), 'm');
hold on;
plot(1:stepSize:numI,cellCountG_noadd(1:stepSize:end), 'c');
%plot(1:numI,cellCountR+cellCountG, 'b');
hold off;
%%
fprintf([num2str(toc(runTime), '%0.1f'), 's\n']);
fprintf('494: creating movies now.\n');
Irgb8 = im2uint8(Irgb);
%Irgb8_f = im2uint8(Irgb_f);
Irgb8_reduced = Irgb8(:,:,:,1:stepSize:end);
fprintf([num2str(toc(runTime), '%0.1f'), 's\n']);
fprintf('499: preparing movie show.\n');
implay(Irgb8,8);
% imshow (Irgb(:,:,:,2));

%%
%save selected variables as mat file

save([fileStruct.basePath, fileStruct.fileNameVar], 'c_V*', 'cell*', '-tabs');

%
%%
% for i = 1:stepSize:numI
```

```
%     i
%     imshow (Irgb(:,:,:,i));
%     hold on;
%     vislabels(L_R_add(:,:,i), 'b',0);
%     vislabels(L_R_add_Removed(:,:,i), 'r',0);
%     drawnow();
%     pause(0.1);
% end
```

5) External function

```
function vislabels(L, color, opt)
%VISLABELS Visualize labels of connected components
%   VISLABELS is used to visualize the output of BWLABEL.
%
%   VISLABELS(L), where L is a label matrix returned by BWLABEL,
%   displays each object's label number on top of the object itself.
%
%   Note: VISLABELS requires the Image Processing Toolbox.
%
%   Example
%   -------
%      bw = imread('text.png');
%      L = bwlabel(bw);
%      vislabels(L)
%      axis([1 70 1 70])

%   Steven L. Eddins
%   Copyright 2008 The MathWorks, Inc.

% Form a grayscale image such that both the background and the
% object pixels are light shades of gray.  This is done so that the
% black text will be visible against both background and foreground
% pixels.

background_shade = 200;
foreground_shade = 240;
I = zeros(size(L), 'uint8');
I(L == 0) = background_shade;
I(L ~= 0) = foreground_shade;

% Display the image, fitting it to the size of the figure.
if opt == 1
    imageHandle = imshow(I, 'InitialMagnification', 'fit');
end

% Get the axes handle containing the image.  Use this handle in the
% remaining code instead of relying on gca.
if opt == 1
    axesHandle = ancestor(imageHandle, 'axes');
else
    axesHandle = gca;
end

% Get the extrema points for each labeled object.
s = regionprops(L, 'Extrema');

% Superimpose the text label at the left-most top extremum location
% for each object.  Turn clipping on so that the text doesn't
% display past the edge of the image when zooming.
hold(axesHandle, 'on');
for k = 1:numel(s)
   e = s(k).Extrema;
   text(e(1,1), e(1,2), sprintf('%d', k), ...
      'Parent', axesHandle, ...
      'Clipping', 'on', ...
```

```
      'Color', color, ...
      'FontWeight', 'bold');
end
hold(axesHandle, 'off');


end
```

6) Utility functions

```
function [I_Dia, I_R, I_G] = adjustImageHistogram(I_Dia, I_R, I_G, numI, stepSize)
% The function adjusts the image histogram by finding
% the minimal and maximal value of the ENTIRE time series

uint16_max = 65535.0;
[sizeXcrop, sizeYcrop] = getImageSize(I_Dia(:,:,1));

% Find minimal and maximal value of ENTIRE time series
minValDia = double(min(min(min(I_Dia(1:stepSize:end)))));
maxValDia = double(max(max(max(I_Dia(1:stepSize:end)))));
minValR = double(min(min(min(I_R(1:stepSize:end)))));
maxValR = double(max(max(max(I_R(1:stepSize:end)))));
minValG = double(min(min(min(I_G(1:stepSize:end)))));
maxValG = double(max(max(max(I_G(1:stepSize:end)))));


%imhist(I_Dia(:,:,1), 2000);
%%
% Adjust each image based on the min/max value found in the time series
for i = 1:stepSize:numI
     %Adjust images by projecting min and max values
     I_Dia(:,:,i) = imadjust(I_Dia(:,:,i),[minValDia/uint16_max; maxValDia/uint16_max],[0.4;1]);
     I_R(:,:,i) = imadjust(I_R(:,:,i),[minValR/uint16_max; maxValR/uint16_max],[0;1]);
     I_G(:,:,i) = imadjust(I_G(:,:,i), [minValG/uint16_max; maxValG/uint16_max],[0;1]);
end


end


function I_bw_cumulated = combine_bwImageSeries(I_bw, operation, numI)
% Combine current mask with previous mask using logical operator
% Each image contains the cummulative information of the previous
% images
% or operation: (0,0)->0 (0,1)->1 (1,0)->1 (1,1)->1

[sizeX, sizeY] = getImageSize(I_bw(:,:,1));
I_bw_cumulated = false(sizeX, sizeY);
I_bw_cumulated(:,:,1) = I_bw(:,:,1); % initialize first image

for i = 2:numI
    if strcmp('or', operation)
        I_bw_cumulated(:,:,i) = or(I_bw(:,:,i), I_bw_cumulated(:,:,i-1));
    elseif strcmp('and', operation)
        I_bw_cumulated(:,:,i) = and(I_bw(:,:,i), I_bw_cumulated(:,:,i-1));
    end
end

end


function Irgb = combineImagesToRGB(I_Dia, IG_BC1, IR_BC1, bwG, bwR, intensityG, intensityR, numI)
% This functions creates a color image from the grey channel images

% I_Dia: Grescale bckground image
```

```
% IG_BC1: Background corrected greyscale green channel image
% IR_BC1: Background corrected greyscale red channel image
% bwG: mask for green channel (is used to remove color outside of cells)
% bwR: mask for red channel (is used to remove color outside of cells)
% intensityG, intensityR: color intensity for final image


Irgb = grey2rgb(I_Dia, numI);

for i = 1:numI
    %greyG = uint16(bwG)*uint16_max; % use this for maximum color

    % green channel
    IG_BC_masked = IG_BC1(:,:,i);
    IG_BC_masked(~bwG(:,:,i)) = 0; % remove color outside of the cells
    H = fspecial('disk',2); % create filter matrix
    greyBlurredG = imfilter(IG_BC_masked,H,'replicate'); % blurr masked green layer
    Irgb(:,:,2,i) = Irgb(:,:,2,i) + greyBlurredG *intensityG;
    %%
    imshow(IG_BC1(:,:,i));
    imshow(bwG(:,:,i));
    imshow(Irgb(:,:,:,i));
    %%
    % red channel
    IR_BC_masked = IR_BC1(:,:,i);
    IR_BC_masked(~bwR(:,:,i)) = 0; % remove color outside of the cells
    H = fspecial('disk',2); % create filter matrix
    greyBlurredR = imfilter(IR_BC_masked,H,'replicate'); % blurr masked green layer
    Irgb(:,:,1,i) = Irgb(:,:,1,i) + greyBlurredR *intensityR;

end

end


function numCells = computeCellList(CellRefLabelFrame, Ibw, numI )
% - This function expects a cell reference frame, wehere all cells of the
% whole time series are cumulated and labeled. Usually the green channel of
% the living cells is used as reference.

% - Ibw contains the green or red channel time series. Each frame is compared to
% the reference frame in order to compute living or dead cells in each
% frame.

% The function returns an array with the number of counted cells

numCells = zeros(numI,1);

for i = 1:numI

%     subplot(1,3,1);
%     vislabels(CellRefLabelFrame,'b',1);
%     subplot(1,3,2);
%     imshow(Ibw(:,:,i))
%     Ibool = and( Ibw(:,:,i), CellRefLabelFrame);
%     subplot(1,3,3);
%     imshow(Ibool);
    cellFrame = CellRefLabelFrame;
    cellFrame(~Ibw(:,:,i)) = 0;
    numCells(i) = numel(unique(cellFrame))-1; % -1: remove count for zero element
end

end


function [I_Dia, I_R, I_G] = cropImages(I_Dia, I_R, I_G, negOffset)
% The function crops the images by according to
```

```
% negOffeset with respect to the right and bottom edge.

%crop images into a quarter of their size
[sizeX, sizeY] = getImageSize(I_Dia(:,:,1));
sizeXcrop = sizeX - negOffset;
sizeYcrop = sizeY - negOffset;

I_Dia(sizeXcrop+1:end,:,:) = [];
I_Dia(:,sizeYcrop+1:end,:) = [];
I_R(sizeXcrop+1:end,:,:) = [];
I_R(:,sizeYcrop+1:end,:) = [];
I_G(sizeXcrop+1:end,:,:) = [];
I_G(:,sizeYcrop+1:end,:) = [];

%I_Dia(:,:,i) = I_Dia(1:sizeXcrop,1:sizeYcrop,i);
%I_Dia2(:,:,i) = I_Dia(1:sizeXcrop,sizeYcrop+1:sizeY,i);
%I_Dia3(:,:,i) = I_Dia(sizeXcrop+1:sizeX,1:sizeYcrop,i);
%I_Dia4(:,:,i) = I_Dia(sizeYcrop+1:sizeX,sizeYcrop+1:sizeY,i);

%I_R(:,:,i) = I_R(1:sizeXcrop,1:sizeYcrop,i);
%I_R2(:,:,i) = I_R(1:sizeXcrop,sizeYcrop+1:sizeY,i);
%I_R3(:,:,i) = I_R(sizeXcrop+1:sizeX,1:sizeYcrop,i);
%I_R4(:,:,i) = I_R(sizeXcrop+1:sizeX,sizeYcrop+1:sizeY,i);

%I_G(:,:,i) = I_G(1:sizeXcrop,1:sizeYcrop,i);
%I_G2(:,:,i) = I_G(1:sizeXcrop,sizeYcrop+1:sizeY,i);
%I_G3(:,:,i) = I_G(sizeXcrop+1:sizeX,1:sizeYcrop,i);
%I_G4(:,:,i) = I_G(sizeXcrop+1:sizeX,sizeYcrop+1:sizeY,i);

end



function D = distMat(v1, v2)
% The function computes the distance matrix for two vectors v1, v2
% For a n1 x 2 vector v1
% and a n2 x 2 vector v2 the matrix gets
% D = n1 x n2

n1 = size(v1,1);
n2 = size(v2,1);

if n1 ~= 0 && n2 ~= 0
    D = zeros(n1,n2);
    for k = 1:n1 % run for all elements of v1
        D(k,:) = sqrt(sum((v1(k*ones(n2,1),:) - v2).^2,2));
    end
else
    D = [];
end

end



function [sizeX, sizeY] = getImageSize(I)
sizeX = size(I,2);
sizeY = size(I,1);
end


function I_bw = grey2bw(I_grey, threshold, minObjectSize, numI)
% This function converts a set of greyscale images to bw images using the
% threshold value.
% Objects which are smaller than minObjectSize are removed from the image
% The images in the set must be two dimensional greyscale (no 3d-rgb)
% The threshold must be defined for each individual image by a threshold
```

```
% array of dimension numI

% Initialize bw image array of the same size
[sizeX, sizeY] = getImageSize(I_grey(:,:,1));
I_bw = false(sizeX,sizeY,numI);

for i = 1:numI
    % compute green binary matrix
    %level = graythresh(Iadj);
    I_temp = im2bw(I_grey(:,:,i),threshold(i));
    I_temp2 = bwareaopen(I_temp, minObjectSize);
    I_bw(:,:,i) = imfill(I_temp2,'holes');

end

end



function I_Dia_rgb = grey2rgb(I_Dia, numI)
% Convert images from 1-channel greyscale to 3-channel rgb
% The appearance dosn't change, because the grey channel is coppied 3 times
[sizeXcrop, sizeYcrop] = getImageSize(I_Dia(:,:,1));
I_Dia_rgb = uint16(zeros(sizeXcrop, sizeYcrop, 3, numI));
for i = 1:numI
    I_Dia_rgb(:,:,:,i) = repmat(I_Dia(:,:,i),[1,1,3]); %replicate three times to generate R, G, B channels
from grayscale
end

end




function I_labeled = label_bwImageSeries(I_bw, numI)
% create labels for the image series

% Initialize bw image array of the same size
[sizeX, sizeY] = getImageSize(I_bw(:,:,1));
I_labeled= int32(zeros([sizeX,sizeY,numI]));

for i = 1:numI
    I_labeled(:,:,i) = bwlabel(I_bw(:,:,i));
end
end



function [I_Dia, I_R, I_G] = loadImages(fileStruct, numI, stepSize)
% The function loads the backround, red and green images determined by
% fileStruct

% Use first image to determine image size
I = imread([fileStruct.basePath, fileStruct.folder_cropped, 'dia\', fileStruct.fileName_Dia, '001.tif']);
sizeX = size(I,2);
sizeY = size(I,1);

% for i_in = 1:stepSize_Dia:numI
%     nameStr_Read = [basePath, folder_cropped, fileName_Dia, num2str(i_in, '%0.3d'), '.tif'];
%     I_Dia = imread(nameStr_Read);
%
% end
% Initialize
I_Dia = uint16(zeros(sizeX, sizeY, numI));
I_R = uint16(zeros(sizeX, sizeY, numI));
I_G = uint16(zeros(sizeX, sizeY, numI));
```

```
% background_R = uint16(zeros(1000, 1000, 19));
% background_G = uint16(zeros(1000, 1000, 19));

% Load images

for i = 1:stepSize:numI
    I_Dia(:,:,i) = imread([fileStruct.basePath, fileStruct.folder_cropped, 'dia\', fileStruct.fileName_Dia, num2str(i, '%0.3d'), '.tif']);
    I_G(:,:,i) = imread([fileStruct.basePath, fileStruct.folder_cropped, 'fitc\', fileStruct.fileName_fitc, num2str(i, '%0.3d'),'.tif']);
    I_R(:,:,i) = imread([fileStruct.basePath, fileStruct.folder_cropped, 'red\', fileStruct.fileName_red, num2str(i, '%0.3d'),'.tif']);
end


end



function [IR_BC, IG_BC] = removeBackground(I_R, I_G, numI, stepSize)
% Compute background for R and G
% Morphologically open image
% Create a disk-shaped structuring element with a radius of 5 pixels.
% se = strel('disk',5);
[sizeXcrop, sizeYcrop] = getImageSize(I_R(:,:,1));

mask_R1 = uint16(zeros(sizeXcrop, sizeYcrop, numI));
mask_G1 = uint16(zeros(sizeXcrop, sizeYcrop, numI));

for i = 1:stepSize:numI
   mask_R1(:,:,i) = imopen(I_R(:,:,i),strel('disk',15));
   mask_G1(:,:,i) = imopen(I_G(:,:,i),strel('disk',15));
end

% Remove background
IR_BC = I_R - mask_R1;
IG_BC = I_G - mask_G1;


end


function I_series = removeBorderElements(L_ref, I_series, edgeFilterSize, numI)
% This function removes the bacteria near the border of an image
% Input arguments:
% L_ref: Label matrix which is used as reference
% I_series: Image or label series where the border elements are removed
% edgeFilterSize: width of border given in pixels

% The function removes the elements from L and M at the same locations
% based on the lables in L and a given edge filter size.

% Output arguments:
% L: Label matrix without removed lables
% M: Matrix where all elements at the same location as in L were removed.
   LBorder = L_ref;
   LBorder(edgeFilterSize:end-edgeFilterSize, ...
      edgeFilterSize:end-edgeFilterSize) =0;
   indexList = unique(LBorder);
   n = numel(indexList);
   for i=1:numI
      for j=1:n
         id = indexList(j);
         I_i = I_series(:,:,i);
         I_i(L_ref == id)=0; %remove elements in all images of the series
         I_series(:,:,i) = I_i;
         %L(L_ref == id)=0; %remove labels
```

```
        end
    end

end



function lRef_out = removeBorderElementsOfRefFrame(L_ref)
% This function removes the bacteria near the border of an image
% Input arguments:
% L_ref: Label matrix which is used as reference
% I_series: Image or label series where the border elements are removed
% edgeFilterSize: width of border given in pixels

% The function removes the elements from L and M at the same locations
% based on the lables in L and a given edge filter size.

% Output arguments:
% L: Label matrix without removed lables
% M: Matrix where all elements at the same location as in L were removed.
    LBorder = L_ref;
    LBorder(edgeFilterSize:end-edgeFilterSize, ...
        edgeFilterSize:end-edgeFilterSize) =0;
    indexList = unique(LBorder);
    n = numel(indexList);
    for j=1:n
      id = indexList(j);
      I_i = I_series(:,:,i);
      I_i(L_ref == id)=0; %remove elements in all images of the series
      I_series(:,:,i) = I_i;
      %L(L_ref == id)=0; %remove labels
    end
end
```
